# SIFT Indel: Predictions for the Functional Effects of Amino Acid Insertions/Deletions in Proteins

**DOI:** 10.1371/journal.pone.0077940

**Published:** 2013-10-23

**Authors:** Jing Hu, Pauline C. Ng

**Affiliations:** 1 Department of Mathematics and Computer Science, Franklin and Marshall College, Lancaster, Pennsylvania, United States of America; 2 Department of Computational and Mathematical Biology, Genome Institute of Singapore, Singapore, Singapore; Ohio State University Medical Center, United States of America

## Abstract

Indels in the coding regions of a gene can either cause frameshifts or amino acid insertions/deletions. Frameshifting indels are indels that have a length that is not divisible by 3 and subsequently cause frameshifts. Indels that have a length divisible by 3 cause amino acid insertions/deletions or block substitutions; we call these 3n indels. The new amino acid changes resulting from 3n indels could potentially affect protein function. Therefore, we construct a SIFT Indel prediction algorithm for 3n indels which achieves 82% accuracy, 81% sensitivity, 82% specificity, 82% precision, 0.63 MCC, and 0.87 AUC by 10-fold cross-validation. We have previously published a prediction algorithm for frameshifting indels. The rules for the prediction of 3n indels are different from the rules for the prediction of frameshifting indels and reflect the biological differences of these two different types of variations. SIFT Indel was applied to human 3n indels from the 1000 Genomes Project and the Exome Sequencing Project. We found that common variants are less likely to be deleterious than rare variants. The SIFT indel prediction algorithm for 3n indels is available at http://sift-dna.org/

## Introduction

Each human genome contains more than ten thousand coding variants [1]. These variants are of considerable interest because most disease variants with severe phenotypic consequences are found in coding regions [2]. The majority of severe diseases are caused by missense mutations (44%) and small coding indels (23%), according to the HGMD database [2]. Missense changes are single base changes that cause an amino acid change in the corresponding protein sequence. Coding indels are insertions or deletions of DNA bases in the coding portion of a gene.

Given the large number of coding variants per genome and their potential importance, the features that distinguish neutral variation from deleterious are of significant interest. For single nucleotide base changes that cause single amino acid substitutions, features such as sequence conservation, predicted structure, and annotation of functional protein regions can help distinguish between neutral and deleterious mutations [3]. These features can be used by prediction algorithms that prioritize the functional effect of a coding variant [3-7]. 

Indels have not been as well characterized as single amino acid substitutions. There are two types of coding indels: those that have lengths that are divisible by 3, and those that do not. Indels with lengths that are not divisible by 3 cause frameshifts, and are presumed to be deleterious to gene function. However, frameshifting indels found in healthy humans tend to occur at the ends of genes or in redundant genes [8-10]. This indicates indel location and gene function are important features for distinguishing between neutral and deleterious frameshifting indels. We use the term “3n indels” for the small indels with lengths divisible by 3. These indels typically lead to an insertion or deletion of amino acids in the corresponding protein sequence. If single amino acid substitutions can affect protein function and play a role in disease, then the insertion/deletion of amino acid(s) could also affect protein function and potentially lead to disease. 3n indels that cause an insertion/deletion of amino acid(s) tend to be in conserved regions and lower disorder regions [11,12]. 

SIFT is a widely used algorithm to predict the effect of missense changes on protein function [13]. SIFT can also predict on frameshifting indels [10]. Here, we extend SIFT by adding prediction for 3n indels that cause insertion/deletion of amino acid(s). In this work, we have constructed a SIFT Indel prediction algorithm which classifies 3n indels as gene-damaging or neutral, and performs comparably to DDIG-in and PROVEAN [11,12]. The purpose of this study is to elucidate important features of deleterious 3n indels, and to provide a prediction method for 3n indels.

## Materials and Methods

### Dataset

The goal of this classifier is to predict if a 3n indel affects gene function (designated as “gene-damaging") or not (designated as “neutral”). The classifier for 3n indels was trained and tested on two datasets:

1. Indel Disease Set: Disease-causing indels were obtained from HGMD [2] version 2010.2. HGMD is a database of mutations found in affected patients, and these mutations are assumed to be gene-damaging. The original HGMD dataset contains 1,887 indels. We picked one indel per gene to be represented in the dataset to avoid over-training on genes that have many HGMD indel entries. After choosing one indel per gene and removing indels from non-exon regions and from genes with invalid/incomplete transcripts, 474 indels remained. The HGMD accession id’s of the 1,887 indels and 474 indels can be found in Data S1.
2. Neutral Indel Set: The set of neutral 3n indels were derived from pairwise alignments from the UCSC genome browser of human with cow, dog, horse, chimpanzee, rhesus macaque and rat [14]. After choosing one indel per gene and removing indels from non-exon regions and from genes with invalid/incomplete transcripts, there were 9,710 neutral indels. The locations of the neutral indels can be found in Data S1.

In addition to the above datasets used for training and test purposes, we use human 3n indels from the 1000 Genomes Project (1K) [15] and the Exome Sequencing Project (ESP) [1] to observe indel trends in the human population. The 1000 Genomes Project is a worldwide collaborative effort to sequence human genomes from different ancestries. As of November 8, 2011, it has over 1000 exomes with at least 20x coverage in 70% of the exomes. The Exome Sequencing Project (ESP) sequenced coding regions of the genome from samples that either served as controls, showed extreme manifestations of specific traits (LDL and blood pressure), or had specific diseases (early onset myocardial infarction and early onset stroke, lung diseases). Despite containing individuals with unfavorable traits, ESP, like dbSNP and 1000 Genomes, is often used as a control and used to filter out neutral variants in order to focus on disease variants [16,17]. For ESP, we used the ESP6500SI dataset which includes 6,503 exomes. 

### Prediction Algorithm

We make a decision tree algorithm for the insertion/deletion of amino acids only. If a 3n indel causes an early stop or frameshift, then this indel is discarded from the training and test set. An example of such an indel is an insertion of TGA which introduces an early stop codon. It will be treated as a framshifting indel and predicted by the SIFT prediction algorithm for frameshifts [10].

If the indel or a part of the indel is repeated in the indel itself and its contiguous flanking sequence, then the indel is deemed to be located in a repeat region.  For example, for the sequence ctcctc-CAT-catctg, the indel will be called as a repeat of (CAT)_2_. Another example is atcgg-CCC-ccacc; since C (an element of the indel) is repeated 5 times in the indel and its flanking sequence, the indel is also considered a repeat of (C)_5_. The minimum length of a repeat is 4 for mononucleotide repeats (e.g. CCCC), and 6 for trinucleotide repeats (e.g. (CAT)_2_). 

Indels in repetitive regions can be described in multiple ways by the genome coordinate system. Because some features in the decision tree algorithm depend on the indel’s location, we wanted a consistent way to describe the location of an indel in the genome. Each indel goes through a preprocessing step for calling consistent genomic coordinates: if an indel resides in a repeat, then the coordinates of the indel will be shifted left before further processing. For example, chr12: 132547088 has an insertion of 3 bases GCA. Because the insertion is flanked by 6 GCA’s 5’ to the indel, the indel coordinates will be adjusted to chr12: 132547070 (Figure S1). Thus, we shift all indel positions to the left if they reside in a repeat.

We used RONN [18] to find the disorder regions of proteins. 

We construct a classifier based on the J48 decision tree algorithm implemented in WEKA[19] to predict if an indel is “gene-damaging” (affects the function of the gene it resides in) or “neutral” (does not affect gene function). We choose to implement the prediction algorithm as a decision tree because it provides interpretable classification rules, which can provide biological insight. Because the dataset is not balanced, we used 474 disease indels and randomly sampled 474 neutral indels from the neutral dataset for training and cross-validation.

### Performance measurement

Ten-fold cross-validation was used to evaluate the performance of the algorithm. The dataset was divided into ten equal-sized subsets. In the ten-fold cross-validation, there were ten rounds of experiments. During each round of experiments, nine subsets were used to train the classifier, and the remaining subset was used to test the classifier. In this study, disease-causing indels are treated as the positive class, while neutral indels are treated as the negative class. True positives (TP) are disease-causing indels predicted as gene-damaging. True negatives (TN) are neutral indels predicted as neutral. False negatives (FN) are disease-causing indels predicted as neutral. False positives (FP) are neutral indels predicted as gene-damaging.

Performance is measured using sensitivity, specificity, precision, accuracy, MCC (Matthews correlation coefficient), and AUC (area under the curve). Sensitivity is the fraction of disease-causing indels that are correctly predicted as gene-damaging. Specificity is the fraction of neutral indels that are correctly predicted. Precision is the percentage of predicted gene-damaging indels that are actually gene-damaging. Accuracy is the percentage of overall predictions that are correct. AUC is the area under the ROC curve when adjusting sensitivity vs. (1-specificity). MCC is a balanced measure of the performance on both positive and negative samples. The formulas for sensitivity, specificity, precision, accuracy and MCC are as follows:

$$sensitivity=\frac{TP}{TP+FN}$$

$$specificity=\frac{TN}{TN+FP}$$

$$precision=\frac{TP}{TP+FP}$$

$$accuracy=\frac{TP+TN}{TP+FN+TN+FP}$$

$$MCC=\frac{TP\times TN-FP\times FN}{\sqrt{\left(TP+FN)(TP+FP)(TN+FP)(TN+FN\right)}}$$

### Feature Selection

For each indel, we have extracted 27 features (see Table S1) describing its properties and influences on the gene product. A heuristic feature selection process was used to choose the features that were useful for the prediction of gene-damaging indels. This feature selection procedure is a simplified version of the Bestfirst method included in WEKA [19]. The search initially started with an empty feature set. Then, one feature was added and 10-fold cross-validation was used to evaluate the performance (i.e., MCC). This step was repeated 27 times so that every feature was attempted individually. After the 27 MCC’s were calculated, the feature with the highest prediction performance was added to the feature set. In the next iteration, every feature from the remaining available features was tested and the feature that showed the largest improvement in the performance (as measured by MCC) when combined with the current selected feature set was chosen to be added to the feature set. The size of feature set was then increased by 1. This feature selection process continued until adding any of the remaining features to the feature set decreased performance. 

## Results

### Motivation for Features in Classifier

For the indels in the disease and neutral datasets, we extracted 27 features describing each indel and the indel’s influence on the gene product (see Table S1). These features were used for training the decision tree algorithm. For example, features describing the physiochemical properties of the amino acids being affected by the indel (e.g. hydrophobicity, volume, mass, surface area, breaking structure) were added as features. The motivation for implementing these features was based on a previous study where Chang and Benner studied protein alignments and looked at the amino acids appearing in and around the gapped regions of the alignment [20]. They found that gapped regions had a propensity for hydrophilic residues but not for hydrophobic residues. Therefore, we implemented amino acid properties as features.

Similarly, we reasoned that amino acids in the secondary structure of the protein (e.g. α-helix, β-sheet) would be important for function, whereas amino acids not in the secondary structure (typically in flexible disordered regions of the protein structure), would not be as important. Therefore, one feature was whether the amino acid that was inserted/deleted was in a disordered region of the protein [15]. 

We were also motivated to identify if an indel is in a repeat region because repetitive indels play a role in polyglutamine diseases [21]. We wanted to be sensitive to indels in small repeats so we implemented a script to detect small exact repeats rather than using the *de facto* algorithm for tandem repeats, Tandem Repeats Finder [22] (see Methods). Six features were from our past frameshifting algorithm [10]; we also added 21 new features (i.e., feature 1, 3-4, 6-7, 12-27) to capture various properties and influences of the 3n indels.

### Performance of the decision tree

Because the number of disease indels and neutral indels is not balanced (~1:20), we used 474 disease indels and randomly sampled 474 neutral indels from the neutral dataset for training and cross-validation to avoid bias toward neutral dataset. The performance of the decision tree trained on this sampled dataset (all 474 disease indels and randomly selected 474 neutral indels) was evaluated using ten-fold cross-validation. We then again sampled a new set of 474 neutral indels from the neutral dataset and the same evaluation process was used. This sampling process was repeated 1,000 times and the average performance and standard deviation were calculated across all samplings. When all 27 features were used, the decision tree achieved an average performance of 80% sensitivity, 80% specificity, 80% precision, 80% accuracy, 0.61 MCC, and 0.82 AUC (Table 1). The standard deviations were within a small range (i.e., 2.0%, 2.0%, 1.7%, 1.5%, 0.03 and 0.02 respectively), which indicates it is reasonable to use one sampling instead of all indels in the neutral dataset for feature selection and training of the final classifier.

**Table 1:** Performance of the decision tree using different features.

Feature used	Sensitivity(%)±SD	Specificity(%)±SD	Precision(%)±SD	Accuracy(%)±SD	MCC±SD	AUC±SD
27 features	80±2.0	80±2.0	80±1.7	80±1.5	0.61±0.03	0.82±0.02
4 features	81	82	82	82	0.63	0.87

SD: Standard deviation

**Figure 1:**
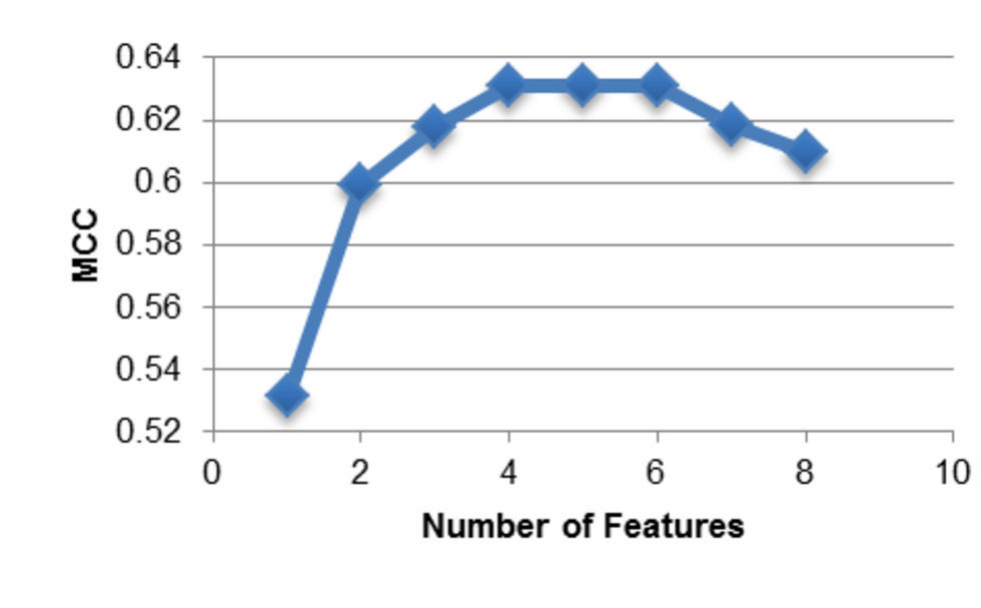
Performance as a function of the number of features. Matthew’s correlation coefficient is used to measure performance. Adding more features beyond the first four features does not substantially improve performance, and can lead to a decline in performance.

We then randomly picked one sampling which consisted of 474 disease indels and 474 neutral indels. We named this dataset the “Balanced Dataset”. We used a greedy feature selection method to search for an optimal set of features that are useful for prediction (see Methods). The final method used four selected features to achieve 82% accuracy, 81% sensitivity, 82% specificity, 82% precision, 0.63 MCC and 0.87 AUC by 10-fold cross-validation (see Table 1). This final method has better performance than using all 27 features (Figure 1). Also, the decision tree and classification rules built from these 4 features are concise, interpretable, and less prone to overtraining. We also applied the final method on the complete dataset (1,887 disease indels and 9,710 neutral indels) and achieved 78% sensitivity, 82% specificity, 45% precision, 82% accuracy, 0.50 MCC, and 0.85 AUC (Table 2). The precision and MCC are low because the large neutral indel dataset causes a high number of false positives which affects these performance metrics. 

**Table 2:** Performances of the decision tree for insertions, deletions and both.

Performance	Balanced Dataset			Complete Dataset		
	Insertions	Deletions	Combined	Insertions	Deletions	Combined
Sensitivity	83% (74 /89)	81% (312/385)	81% (386/474)	91% (257/283)	76% (1154 /1526)	78% (1411/1809*)
Specificity	82% (172 / 211)	82% (215/263)	82% (387/474)	84% (3477/4125)	81% (4501/5585)	82% (7978/9710)
Accuracy	82% (246 /300 )	82% (527/648)	82% (773/948)	85% (3477/4408)	80% (5655/7111)	82% (9389/11519)

* Out of 1,887 disease indels, 1,809 have predictions. The rest lack of predictions because they are located in genes with no valid/complete transcript information.

**Figure 2:**
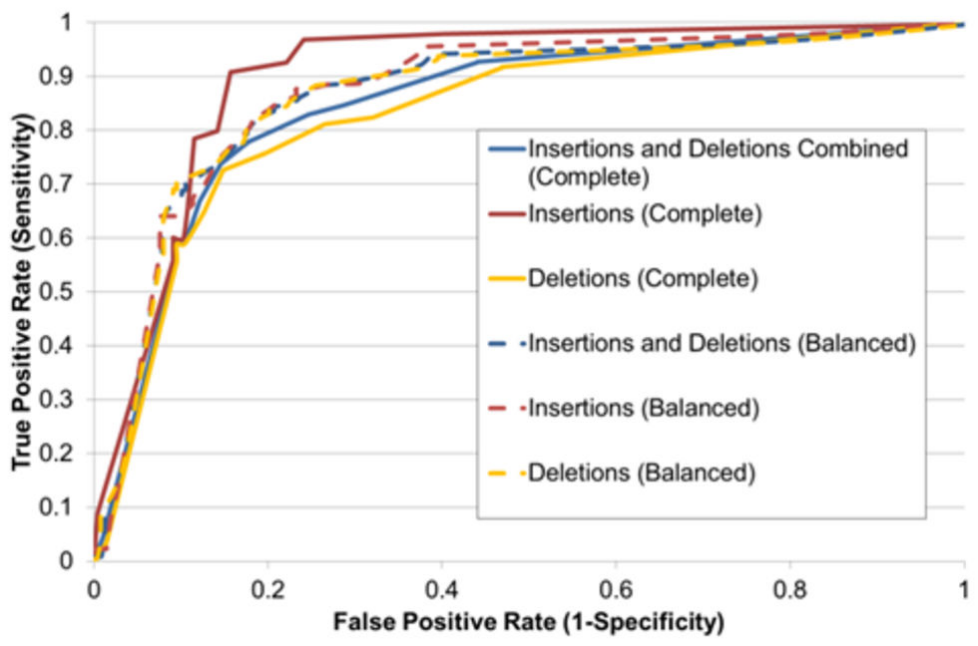
SIFT ROC curves for insertions and deletions combined, insertions only, and deletions only. The balanced dataset is the training set for the decision tree and consists of 474 disease indels and 474 neutral indels. The complete dataset consists of 1,809 disease indels and 9,710 neutral indels.

We were also interested in the performance of the decision tree for insertions and deletions separately. The performances of 10-fold cross-validations are similar for insertions and deletions combined, insertions only, and deletions only on the balanced dataset used for training (Table 2). We also applied the final decision tree to predict on the complete dataset. For this larger dataset, the decision tree achieved better accuracy on insertions than deletions (85% vs. 80%) (Table 2). For comparison, we also plot the ROC curves of predictions on the balanced and complete datasets for insertions and deletions combined, insertions only, and deletions only (Figure 2). For the balanced dataset, the AUC is 0.87 for insertions and deletions combined, 0.87 for insertions only, and 0.87 for deletions only. For the complete dataset, the AUC is 0.85 for insertions and deletions combined, 0.90 for insertions only, and 0.83 for deletions only. The decision tree achieved better performances on insertions than deletions. However, if we require high specificities (specificity>90%, or FPR<0.1), then sensitivities are similar for all 6 datasets (see overlap of all curves on left side of Figure 2). 

Insertions have higher performance compared to deletions, and this was also found in PROVEAN [12]. DDIG-in initially trained insertions and deletions separately, but the final SVM was trained on the combined set of insertions and deletions because of the large overlap of features [11]. For our decision tree, we decided to combine insertions and deletions as a single dataset for training of the decision tree due to the small training dataset, especially for insertions. Widespread sequencing will identify additional indels so that training on larger datasets in the future may stabilize performance for insertions. 

### Analysis of selected features and trained classification rules

The four features for prediction were chosen using the heuristic feature selection process described in the Feature Selection section of Methods. These features, in the order they were chosen, are: 1) fraction of Pfam [23] domains affected, 2) whether the indel resides in a repeat, 3) whether the indel is in a disordered region [24],and 4) the conservation score of the DNA base to the left of the allele obtained from PhyloP [25]. They are features 9, 1, 27 and 3 as described in Table S1.

There are 10 classification rules derived from the trained decision tree (see Table S2). A classification rule is extracted by following a path from the root of the decision tree to one of its leaves. The class of most training samples which follow the path gives a classification rule; while the percentage of training samples which matches the class of the leaf on that path gives the confidence score of such classification. Three rules can be used to predict on the majority of the training indels (i.e., almost 60% of gene-damaging indels and 84% of neutral indels). These rules are as follows:

Rule 4: If no Pfam domain is affected, the indel is not in a repeat but in the disordered region of gene’s protein product, then the indel will not affect the gene function. The confidence score of this rule is 0.918. (Out of 317 training samples that followed this classification rule, 291 of them were actually functional neutral.)

Rule 5: If no Pfam domain is affected, the indel is not a repeat and is not located in the disordered region of the protein product, and the DNA base 5’ of the allele is not conserved (conservation score ≤ 1.405), then the indel will be functionally neutral with a confidence score of 0.720. (Out of 82 training samples that followed this classification rule, 59 of them were actually functionally neutral.)

Rule 10: If there are Pfam domains affected and the indel is not located in disordered region, then the indel will be gene-damaging with a confidence score of 0.894. (Out of 284 training samples that followed this rule, 254 of them actually affect gene functions.) The biological reasoning for this rule is that if the indel is not in a disordered region then it is located in secondary structure which also has functionality according to Pfam. Thus, indels that disrupt structure/function are damaging to gene function.

Because rules 4,5, and 10 cover the majority of the training set, we also reported the performance of 10-fold cross-validation by using combinations of just these three rules (see Table S3).

Together these rules reflect that an indel is unlikely to affect gene function if it does not reside in a Pfam functional domain, is not a repeat, resides in a disordered region, and/or its left flanking base is not conserved. An indel is more likely to be gene-damaging if the indel affects Pfam domains, is a repeat, not in disordered region, and/or its left flanking DNA base is highly conserved. 

**Figure 3:**
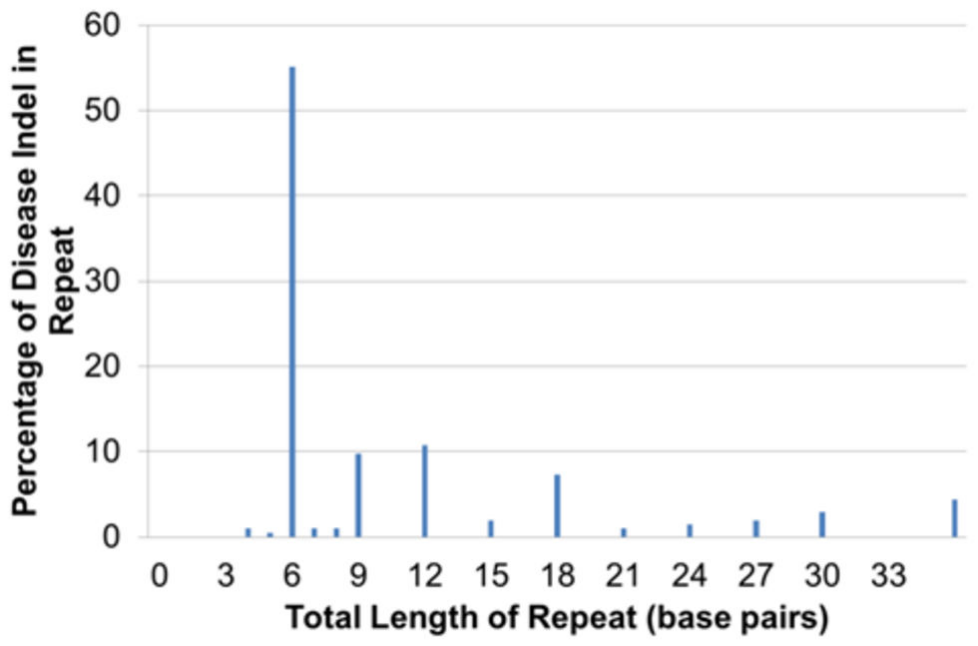
Repeat length distribution for disease indels in small exact repeat regions. On the x-axis is the total length of the repeat, which is the product of the period size of the repeat and the number of copies of the repeat.

Disease indels are 4 times more likely to occur in repeats than neutral indels; 11% of neutral indels versus 43% of disease indels appear in repeat regions. The period size of the repeat tends to be divisible by 3, consistent with a repeating amino acid or amino acids (Figure 3). The number of copies of the repeat for disease indels can be small. Half of the disease indels are due to two copies of a trinucleotide repeat (e.g. (ACG)_2_) (Figure 3). This indicates that disease indels are frequently due to an insertion that is a simple duplicate of an amino acid (e.g. CTT → (CTT)_2_ causes Leu → LeuLeu) or a deletion of an amino acid from 2 amino acids with 2 identical codons (e.g. (AAG)_2_ → AAG causes LysLys → Lys). Polymerase slippage causes indels to occur in sequence that is repeated [26]. Slippage is a frequent mutation mechanism, and slippage-like indels are known to occur 5-9x more frequently than random mutations [27]. Hence, a substantial percentage of the disease indels are due to intrinsic DNA mutation susceptibilities. 

### Prediction on Human Indels

 Evolution selects against deleterious variants. Consequentially, common variants are less likely to be deleterious than rare variants, and this has been confirmed for missense substitutions and frameshifting indels [3,4]. We applied the SIFT Indel algorithm to the 3n indels identified from the 1000 Genomes Project (1K) [15] and the Exome Sequencing Project (ESP) [1], and observed similar trends for 3n indels. We calculated the fraction of indels that were predicted damaging for indels identified by 1000 Genomes and the Exome Sequencing Project. As expected and across all datasets, a higher fraction of rare indels were predicted damaging compared to common indels (Table 3). Over 50% of the rare 3n indels were predicted damaging (MAF <= 0.05). For common 3n indels (MAF > 0.05), the percentage predicted damaging ranged from 30% to 45%, and this appears to be highly dependent on the source of the data. Common indels from Exome Sequencing Project were more likely to be predicted tolerated than common indels from 1000 Genomes Project, which may due to differences between the data sets with different sequencing technologies and downstream variant calling pipelines. 

**Table 3:** Percentage of 3n indels predicted damaging, according to allele frequency and population.

	Exome Sequencing Project		1000 Genomes		
	EUR	AFR	EUR	ASN	AFR
Rare (MAF <= 0.05)	55%	55%	54%	57%	52%
Common (MAF > 0.05)	30%	30%	41%	49%	46%

EUR: European, AFR: African, ASN: Asian

**Figure 4:**
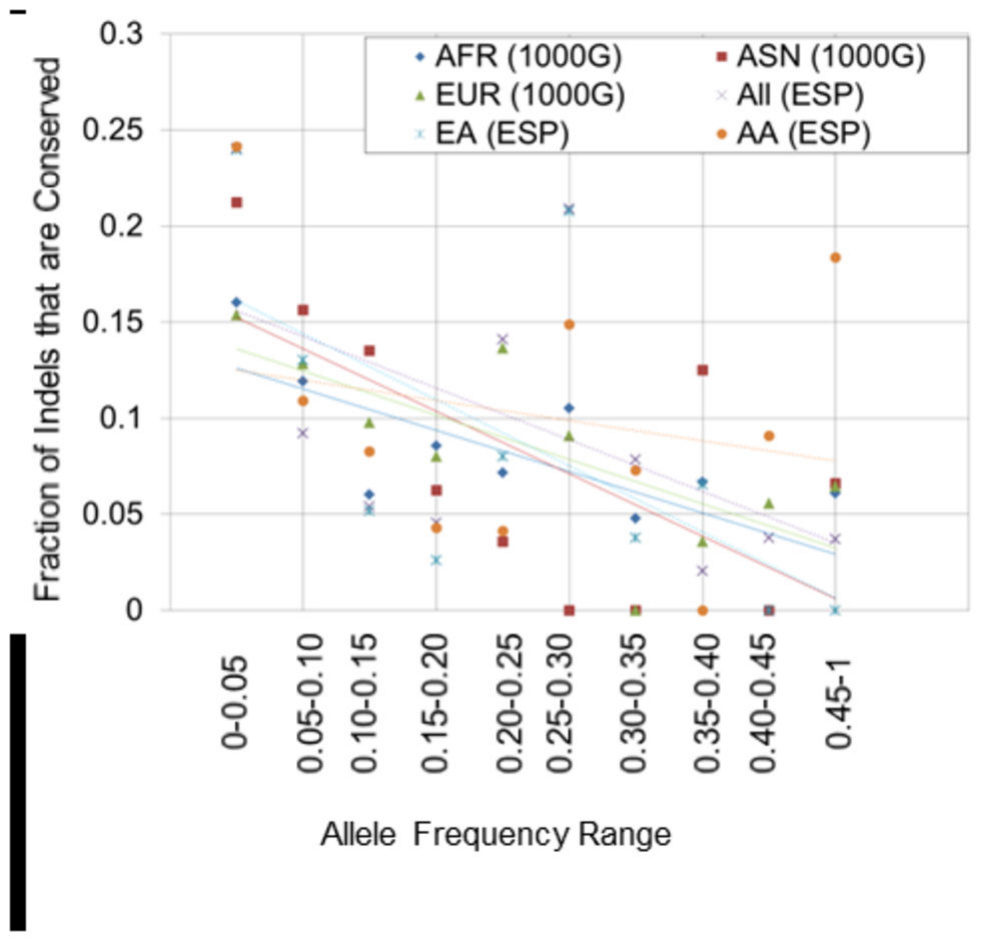
The fraction of indels that are conserved for indels in a given allele frequency range. Conserved is defined as a GERP score > 2. 1000G: 1000 Genomes; AFR: African; EUR: European; ASN: Asian; ESP: Exome Sequencing Project; EA: European American; AA: African American.

We examined the relationship between indels’ allele frequencies and DNA conservation. For a given range of allele frequencies (e.g. Minor allele frequency =0.00- 0.05), we calculated the fraction that of indels that are conserved, as defined by having a GERP conservation score > 2 [25]. Rare indels were more likely to be conserved than common indels (Figure 4). 

## Discussion

We have previously shown that SIFT predicts on amino acid substitutions and frameshifting indels. Here, we enhance SIFT to predict on short insertions/deletions of amino acid substitutions. 

### Biological Insights from the Decision Tree

One would expect that insertion/deletion of amino acids in a functional region to alter gene function. This is consistent with our decision tree’s rule that when an indel resides in a Pfam domain, the indel is more likely to be damaging. Similarly, disordered regions of a protein evolve more rapidly than the ordered regions [28], so it is not surprising that disordered regions can withstand variation; therefore, indels in these regions would be predicted to be neutral.

The conservation score of the DNA base 5’ to the indel was another feature selected, where low conservation at the base returns a neutral prediction. If the conservation score of the DNA base 5’ of the indel is low, then this could indicate the indel is flanking the third base of a codon. The third base of a codon can be degenerate (e.g. ACN encodes Threonine) and tends not to be conserved. This suggests the indel coincides with codon boundaries and amino acid(s) would be precisely inserted or deleted, which is a phase 0 indel event and no amino acid changes occur in the flanking sequence [27]. Our algorithm suggests that precise insertion/deletion of amino acids have less deleterious consequences on gene function than 3n indels that change neighboring amino acids in addition to inserting/deleting amino acids.

### Comparison with Other Tools

Recently two other tools which can predict the effects of 3n indels have been published. PROVEAN [11] predicts the effect of 3n indels by measuring the change of sequence similarity scores of the target protein with its homologous proteins. It achieved 82% accuracy for deletions and 87% accuracy for insertions on human indels extracted from UniProt’s “Human Polymorphisms and Disease Mutations” dataset. DDIG-in [12] is a support vector machine-based method by exploring features at both nucleotide and protein level, including DNA conservation scores and disorder scores. Using selected features, DDIG-in yielded 0.684 for MCC, 85% for accuracy and 0.89 for AUC on dataset of both insertions and deletions by 10-fold cross-validation. Therefore, our prediction tool has similar accuracy (82%) compared to PROVEAN (82%) and DDIG-in (85%).

Training datasets determine how an algorithm is constructed and performs. DDIG-in and SIFT 3n Indel used disease indels from HGMD, but different neutral datasets. DDIG-in’s neutral dataset were indels from healthy individuals sequenced by 1000 Genomes Project. Our motivation for using cross-species comparisons for our neutral dataset was because indels from apparently healthy human individuals might still damage gene function yet not manifest as disease. Indels from cross-species comparisons have undergone millions of years of selection, and the majority of variants with small negative selection coefficients should have been eliminated. Despite the differences in datasets, both DDIG-in and SIFT have selected similar features for being important for prediction, such as conservation and disorder scores.

### Comparison of rules for 3n Indels and Frameshifting indels

The rules for the prediction of 3n indels conform to known biological insights about insertions/deletions of amino acids in protein sequence. We compare the rules for amino acid insertion/deletion prediction with the rules extracted for frameshifting indels in our previous study [10]. For both frameshifting and amino acid indels, conservation is important. However, conservation is considered at the direct location of the insertion/deletion of the amino acid for 3n indels. In contrast, for frameshifting indels, conservation is important downstream of the indel location, because this indicates if protein function has been lost as a result of the frameshift. In general, 3n indels affect the local environment surrounding the mutation, while frameshifting indels affect everything downstream of the indel. 

Amino acid changes and coding indels encompass over two-thirds of known disease mutations [2]. Thus, SIFT supplies predictions for the two largest classes of known disease mutations. These types of prediction methods will aid researchers in prioritizing newly discovered variation. 

## Supporting Information

Table S1 **List of all features tested by the decision tree.**
(DOCX)

Table S2 **Classification rules for prediction.**
(DOCX)

Table S3 **Performance of 10-fold cross-validations using subsets of rules.**
(DOCX)

Figure S1 **Shifting indels in repeats to the leftmost position.** An indel has a position assigned at chr12:132,547,088 (filled red arrow). Because the indel is in a repeat sequence, it could also be assigned other locations and still result in the same DNA change (dashed purple arrows). Due to the ambiguity of locations, we assign indels in repeats the leftmost position (chr12:132,547,070, filled purple arrow).
(TIF)

Data S1 **List of neutral indel locations and HGMD id’s of disease indels.**
(XLSX)
