# Supplementary material for: SIFT Indel: Predictions for the Functional Effects of Amino Acid Insertions/Deletions in Proteins
Source: PLoS One. 2013 Oct 23;8(10):e77940. doi: 10.1371/journal.pone.0077940 (PMC3806772; doi:10.1371/journal.pone.0077940)
Supplement: Table S1 — List of all features tested by the decision tree. (DOCX) [file pone.0077940.s001.docx]

Table S1. List of all features tested by the decision tree; the features used in the final decision tree are highlighted in red.

| Feature Number | Feature Name | Feature description |
| --- | --- | --- |
| 1 | Whether the indel is a repeat or not. | Compare the indel with its flanking sequence to see if the indel is a repeat. |
| 2 | Maximum relative indel location | For each affected transcript of the affected gene, calculate the relative indel position as position of indel on the coding sequence divided by the length of coding sequence. Take the maximum relative indel location across all transcripts for the affected gene. |
| 3 | DNA conservation score of the nucleotide to the left of the indel | The conservation score of each DNA base is obtained from PhyloP [[1](#_ENREF_1)]. A high positive score indicates the base is conserved, a negative score indicates positive selection, and a 0 score represents neutral selection. For each indel, the conservation score of the nucleotide to the left of the indel is extracted. |
| 4 | DNA conservation score of the nucleotide to the right of the indel | Same as 3, except the conservation score of the nucleotide to the right of the indel is extracted. |
| 5 | Minimum distance of indel to the exon boundary of all affected transcripts | For all affected transcripts, calculate the minimum distance of indel to the exon boundary. |
| 6 | The conservation score of the amino acid to the left of the indel | To calculate conservation scores of amino acids of the translated protein, we followed the SIFT method for choosing sequences [[2](#_ENREF_2)] by searching a database of proteins from vertebrate genomes. The SIFT procedure generates a protein sequence alignment, conservation values were calculated for each position [[3](#_ENREF_3)]. Then the conservation score of the amino acid to the left of the indel position is extracted. |
| 7 | The conservation score of the amino acid to the right of the indel | Same as 6, except the conservation score of the amino acid to the right of the indel position is extracted. |
| 8 | Fraction of all functional domains (Pfam, super family, signal peptide, Seg, ncoils, TMHMM, etc.) affected due to indel. | Functional domains of each protein are downloaded from Ensembl [[4](#_ENREF_4)]. For each affected transcript, calculate the percentage of all functional domains as annotated by Ensembl, including Pfam domains, super family domains, signal peptides, and all other domains lost from the newly translated protein due to indel. Then for all the affected transcripts, calculate the average fraction. |
| 9 | Fraction of all Pfam domains affected due to indel. | Same as 8, but restricted to Pfam domains. |
| 10 | Fraction of all super family domains affected due to indel. | Same as 8, but restricted to super family domains. |
| 11 | Fraction of all signal peptide domains affected due to indel. | Same as 8, but restricted to signal peptide domains. |
| 12 | Average mass of the amino acids at the indel position | The mass values of amino acids are obtained from the Amino Acid Repository (http://jenalib.fli-leibniz.de/IMAGE_AA.html).Calculate the average mass of all amino acids at the indel position. |
| 13 | Average mass of the amino acids to the left of indel | Same as 12, but restricted to left flanking sequence (<=5 amino acids) of the indel position. |
| 14 | Average mass of the amino acids to the right of indel | Same as 12, but restricted to right flanking sequence (<=5 amino acids) of the indel position. |
| 15 | Average surface area of the amino acids of the indel | The surface area values of amino acids are obtained from the Amino Acid Repository (http://jenalib.fli-leibniz.de/IMAGE_AA.html). Calculate the average surface area of all amino acids at the indel position. |
| 16 | Average surface area of the amino acids to the left of indel | Same as 15, but restricted to left flanking sequence (<=5 amino acids) of the indel position. |
| 17 | Average surface area of the amino acids to the right of indel | Same as 15, but restricted to right flanking sequence (<=5 amino acids) of the indel position. |
| 18 | Average volume of the amino acids of the indel position | The volume values of amino acids are obtained from the Amino Acid Repository (http://jenalib.fli-leibniz.de/IMAGE_AA.html). Calculate the average surface area of all amino acids at the indel position. |
| 19 | Average volume of the amino acids to the left of indel | Same as 18, but restricted to left flanking sequence (<=5 amino acids) of the indel position. |
| 20 | Average volume of the amino acids to the right of indel | Same as 18, but restricted to right flanking sequence (<=5 amino acids) of the indel position. |
| 21 | Whether amino acids at the indel positions have structure breaking amino acids | Check to see if amino acids at indel positions have any classic structure-breaking amino acids (P, G, D, S)^a^. |
| 22 | Whether amino acids on the left flanking sequence of the indel have structure breaking amino acids | Same as 21, but restricted to left flanking sequence (<=5 amino acids) of the indel position. |
| 23 | Whether amino acids on the right flanking sequence of the indel have structure breaking amino acids | Same as 21, but restricted to right flanking sequence (<=5 amino acids) of the indel position. |
| 24 | Whether amino acids on the indel positions have hydrophilic amino acids | Check to see if amino acids at indel positions have any hydrophilic amino acids (A, Q, E)^a^. |
| 25 | Whether amino acids on the left flanking sequence of the indel have hydrophilic amino acids | Same as 24, but restricted to left flanking sequence (<=5 amino acids) of the indel position. |
| 26 | Whether amino acids on the right flanking sequence of the indel have hydrophilic amino acids | Same as 24, but restricted to right flanking sequence (<=5 amino acids) of the indel position. |
| 27 | Whether the indel is located in protein disorder region | RONN [[5](#_ENREF_5)] is used to calculate the disorder score (in the range of 0-1) of each amino acid on proteins. If the disorder score of an amino acid is greater than 0.5, then it is considered to be in disorder region. |

a. Previous studies comparing insertions and deletions in coding regions

between multiple species have shown that certain amino acids and

certain regions in proteins are prone to indels. Chang and Benner

studied protein alignments and looked at the amino acids appearing in

and around the gapped regions of the alignment [[6](#_ENREF_6)]. They found that gapped regions have a propensity for hydrophilic residues (AQE) and

classic structure-breaking amino acids (P,G,D,S), but not for

hydrophobic residues.

**References**

1. Siepel A, Bejerano G, Pedersen JS, Hinrichs AS, Hou M, et al. (2005) Evolutionarily conserved elements in vertebrate, insect, worm, and yeast genomes. Genome Res 15: 1034-1050.

2. Ng PC, Henikoff S (2002) Accounting for human polymorphisms predicted to affect protein function. Genome Res 12: 436-446.

3. Schneider TD, Stormo GD, Gold L, Ehrenfeucht A (1986) Information content of binding sites on nucleotide sequences. J Mol Biol 188: 415-431.

4. Flicek P, Aken BL, Ballester B, Beal K, Bragin E, et al. (2010) Ensembl's 10th year. Nucleic Acids Res 38: D557-562.

5. Yang ZR, Thomson R, McNeil P, Esnouf RM (2005) RONN: the bio-basis function neural network technique applied to the detection of natively disordered regions in proteins. Bioinformatics 21: 3369-3376.

6. Chang MS, Benner SA (2004) Empirical analysis of protein insertions and deletions determining parameters for the correct placement of gaps in protein sequence alignments. J Mol Biol 341: 617-631.
