# Supplementary material for: SIFT Indel: Predictions for the Functional Effects of Amino Acid Insertions/Deletions in Proteins
Source: PLoS One. 2013 Oct 23;8(10):e77940. doi: 10.1371/journal.pone.0077940 (PMC3806772; doi:10.1371/journal.pone.0077940)
Supplement: Table S2 — Classification rules for prediction. (DOCX) [file pone.0077940.s002.docx]

Table S2.Classification paths extracted from the decision tree for predicting 3n indels.

| Rule | Fraction of Pfam domains affected | Whether the indel resides in a repeat | Whether the indel is in a disordered region | The conservation score of the DNA base to the left of the allelle | Prediction | Confidence (a/b) |
| --- | --- | --- | --- | --- | --- | --- |
| 1 | 0 | Yes | Yes | <=0.301 | Neutral | 0.696 (16/23) |
| 2 | 0 | Yes | Yes | >0.301 | Damaging | 0.667 (32/48) |
| 3 | 0 | Yes | No |  | Damaging | 0.826 (38/46) |
| 4 | 0 | No | Yes |  | Neutral | 0.918 (291/317) |
| 5 | 0 | No | No | <=1.405 | Neutral | 0.720 (59/82) |
| 6 | 0 | No | No | >1.405 | Damaging | 0.765 (26/34) |
| 7 | >0 | Yes | Yes |  | Damaging | 0.943 (33/35) |
| 8 | >0 | No | Yes | <=2.058 | Neutral | 0.636(42/66) |
| 9 | >0 | No | Yes | >2.058 | Damaging | 0.846 (11/13) |
| 10 | >0 | Yes or No | No |  | Damaging | 0.894 (254/284) |

1. Number of correct samples in the training dataset that follow this rule.
2. Number of all samples in the training dataset that follow this rule.
