# Supplementary material for: SIFT Indel: Predictions for the Functional Effects of Amino Acid Insertions/Deletions in Proteins
Source: PLoS One. 2013 Oct 23;8(10):e77940. doi: 10.1371/journal.pone.0077940 (PMC3806772; doi:10.1371/journal.pone.0077940)
Supplement: Table S3 — Performance of 10-fold cross-validations using subsets of rules. (DOCX) [file pone.0077940.s003.docx]

Table S3. Performance of 10-fold cross-validations using subsets of rules, specifically for the combination of Rules 4 + 10, and Rules 5 + 10.

| Principles | Sensitivity | Specificity | Precision | Accuracy | MCC |
| --- | --- | --- | --- | --- | --- |
| Rule 4+Rule 10 | 89 | 92 | 91 | 91 | 0.81 |
| Rule 5 + Rule 10 | 89 | 72 | 92 | 86 | 0.60 |

Rule 4: If no Pfam domain is affected, the indel is not in a repeat but in the disordered region of gene’s protein product, then the indel will not affect the gene function.

Rule 5: If no Pfam domain is affected, the indel is not a repeat and is not located in the disordered region of the protein product, and the DNA base 5’ of the allele is not conserved (conservation score ≤ 1.405), then the indel will be functionally neutral.

Rule 10: If there are Pfam domains affected and the indel is not located in disordered region, then the indel will be gene-damaging.

Since each rule extracted from the decision tree only tests certain value range of each feature, each rule can only cover a fraction of the whole dataset. Rules 4 and 10 only cover 63.4% (601 out of 948 indels) of the training set of Decision Tree. Rules 5+10 only cover 38.6% (366 out of 948 indels) of the training set of Decision Tree
