# Supplementary figures and images for: SIFT Indel: Predictions for the Functional Effects of Amino Acid Insertions/Deletions in Proteins
Source: PLoS One. 2013 Oct 23;8(10):e77940. doi: 10.1371/journal.pone.0077940 (PMC3806772; doi:10.1371/journal.pone.0077940)

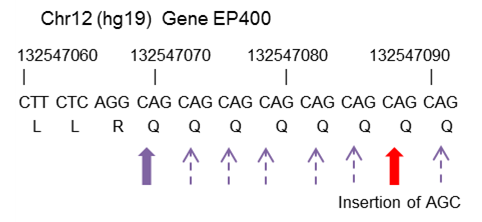

Supplement: Figure S1 — Shifting indels in repeats to the leftmost position. An indel has a position assigned at chr12:132,547,088 (filled red arrow). Because the indel is in a repeat sequence, it could also be assigned other locations and still result in the same DNA change (dashed purple arrows). Due to the ambiguity of locations, we assign indels in repeats the leftmost position (chr12:132,547,070, filled purple arrow). (TIF) [file pone.0077940.s004.tif]
